# Stochastic Resonance and Safe Basin of Single-Walled Carbon Nanotubes with Strongly Nonlinear Stiffness under Random Magnetic Field

**DOI:** 10.3390/nano8050298

**Published:** 2018-05-04

**Authors:** Jia Xu, Chao Li, Yiran Li, Chee Wah Lim, Zhiwen Zhu

**Affiliations:** 1 Department of Mechanics, Tianjin University, Tianjin 300072, China; xujia@tju.edu.cn (J.X.); lichaotju@163.com (C.L.); liyirantju@163.com (Y.L.); 2 Department of Architecture and Civil Engineering, City University of Hong Kong, Tat Chee Avenue, Kowloon, Hong Kong 999077, China; bccwlim@cityu.edu.hk; 3 Tianjin Key Laboratory of Nonlinear Dynamics and Control, Tianjin University, Tianjin 300072, China

**Keywords:** random magnetic field, safe basin, single-walled carbon nanotubes, stochastic resonance, strong nonlinearity

## Abstract

In this paper, a kind of single-walled carbon nanotube nonlinear model is developed and the strongly nonlinear dynamic characteristics of such carbon nanotubes subjected to random magnetic field are studied. The nonlocal effect of the microstructure is considered based on Eringen’s differential constitutive model. The natural frequency of the strongly nonlinear dynamic system is obtained by the energy function method, the drift coefficient and the diffusion coefficient are verified. The stationary probability density function of the system dynamic response is given and the fractal boundary of the safe basin is provided. Theoretical analysis and numerical simulation show that stochastic resonance occurs when varying the random magnetic field intensity. The boundary of safe basin has fractal characteristics and the area of safe basin decreases when the intensity of the magnetic field permeability increases.

## 1. Introduction

With the advancement of the nanotechnology, carbon nanotubes (CNTs) have been now among the most promising components in nanoelectromechanical systems (NEMS). In recent years, CNTs have attracted worldwide attention because of their potential applications in many areas of science and engineering such as electronics, chemistry, nanoengineering, materials science, thermal and other physical attributions [1,2,3]. They have been widely used in NEMS, for example in nanobiological devices. In order to obtain a good understanding of CNTs and to design new nanodevices, it is very important to build more accurate theoretical models and to analyze their properties.

For these nanostructures at such minute scales, the classical (local) continuum mechanics models are deemed to fail because the classical models not only disregard surface and size effects but also assume the stress state at a given point to depend uniquely on the strain state at that identical point. Since the early 1970s, Eringen [4,5] proposed the nonlocal elasticity theory on the assumption that the stress at a point in a domain depends not only on the classical local stress at that particular point but also on the spatial integrals that represent the weighted averages of the local stress contribution of all other points in the domain.

When correctly formulated by adding the constitutive boundary conditions, the resulting continuous nonlocal elastostatic problem of a bounded nanostructure becomes ill-posed [6], due to conflicting constitutive and equilibrium conditions on the stress field [7]. Consequently, no solution of the nonlocal structural problem does exist [8] and this is the reason why paradoxical results are reported in literature [9,10]. This serious drawback can be overcome by adopting the innovative stress-driven nonlocal integral model, where the roles of stress and elastic strain fields are swapped [11]. The stress-driven nonlocal model has been successfully adopted in several papers [12,13,14].

Subsequently, much progress on nonlinear problems of nanostructures with the nonlocal elasticity theory has been reported [15,16,17,18,19,20,21,22,23,24,25,26,27,28,29,30,31,32,33,34]. The deformation and vibration of nanobeams have been studied by Lim [15,16] and Reddy [17], the assessment of nanotube structures has been investigated by Kiani, K. [18]. The studies on buckling of nanotubes have been reported in various references [19,20,21]. In addition, plenty of research results on nanotubes vibration have been reported recently [22,23,24,25,26,27,28,29,30,31,32,33,34,35,36], for example Soltani analyses the nonlinear free and forced vibration of a single-walled carbon nanotube using shell model [34], Manevitch studies the nonlinear optical vibrations of single-walled carbon nanotubes [35], Ouakad researches the nonlinear dynamics of electrically actuated carbon nanotube resonators [36].

For the small size of NEMS, not only the nonlocal effect must be considered but also the strongly nonlinearity and random factors cannot be ignored. Recently, several researches have been reported on random response of carbon nanotubes [37,38,39,40,41,42]. The current paper aims to provide a kind of method to study the strongly nonlinear dynamical characteristics of CNTs subjected to random magnetic field. Considering the nonlocal effect, the nonlinear dynamic model of single-walled carbon nanotubes subjected to random magnetic field is established. The natural frequency of the strongly nonlinear dynamic system is obtained by the improved energy function method and stochastic dynamical characteristics of the system are analyzed.

## 2. Strongly Nonlinear Model of Single-Walled Carbon Nanotubes

**Figure 1:**
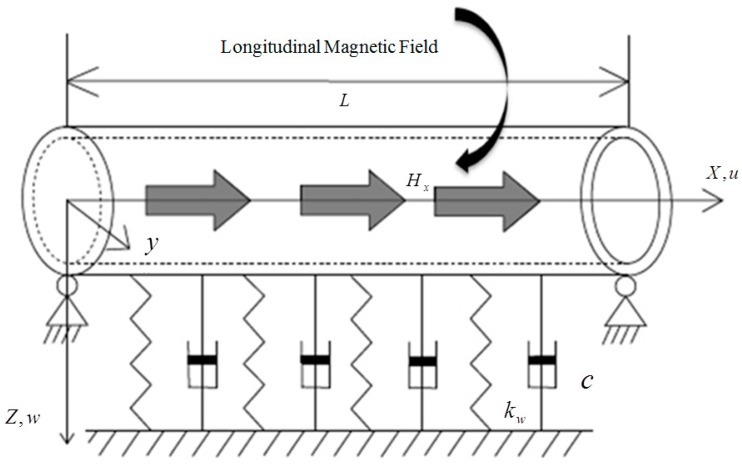
Mechanical model of single-walled carbon nanotubes.

As shown in Figure 1, the mechanical model of a single-walled carbon nanotube is modeled as a simply supported pipe at both ends. The length of tube is L, the spring is $k_{w}$, the damper is c and the longitudinal magnetic field is $H_{x}$.

According to the results of references [43,44], the dynamic model of a single-walled carbon nanotube subjected to a longitudinal magnetic field can be modelled as follows:

(1) $$\begin{array}{l} EI\frac{\partial^{4}w}{\partial x^{4}}+\frac{E\bar{A}}{2L}\left[\int_{0}^{L}{\left(\frac{\partial w}{\partial x}\right)}^{2}dx\right]\frac{\partial^{2}}{\partial x^{2}}\left[{\left(e_{0}a\right)}^{2}\frac{\partial^{2}w}{\partial x^{2}}-w\right]-\bar{f}\left(x,t\right)+{\left(e_{0}a\right)}^{2}\frac{\partial^{2}\bar{f}}{\partial x^{2}}=\\ m\frac{\partial^{2}}{\partial t^{2}}\left[{\left(e_{0}a\right)}^{2}\frac{\partial^{2}w}{\partial x^{2}}-w\right]+k_{w}\left[{\left(e_{0}a\right)}^{2}\frac{\partial^{2}w}{\partial x^{2}}-w\right]+c_{1}\frac{\partial }{\partial t}\left[{\left(e_{0}a\right)}^{2}\frac{\partial^{2}w}{\partial x^{2}}-w\right]+c_{3}\frac{\partial }{\partial t}{\left[{\left(e_{0}a\right)}^{2}\frac{\partial^{2}w}{\partial x^{2}}-w\right]}^{3} \end{array}$$

where E is the Young’s modulus, I is the second moment of area, $\bar{A}$ is the cross sectional area, $e_{0}$ is a constant appropriate to each material, a is an internal characteristic length, m is the mass, $c_{1}$ is the linear damper coefficient, $c_{3}$ is the cubic nonlinear damper coefficient, $\bar{f}\left(x,t\right)=\xi \bar{A}H_{x}\frac{\partial^{2}w}{\partial x^{2}}$, ξ is the magnetic field permeability and w(x,t) is the displacement function of the system.

There always exist random disturbances in the system. The longitudinal magnetic field $H_{x}$ in this paper is considered as, more realistically, a stochastic magnetic field and $H_{x}=H-B\left(t\right)$, H is the deterministic magnetic field intensity, B(t) is Gauss white noise whose mean is zero and intensity is 2D (D>0). Let $w\left(x,t\right)=u\left(t\right)\sin\frac{\pi x}{L}$, we can obtain the dynamic equation from Equation (1) by Galerkin’s method as follows

(2) $$\ddot{u}+\frac{k_{w}l^{4}\alpha +\pi^{2}\xi \bar{A}Hl^{2}\alpha +\pi^{4}EI}{ml^{4}\alpha }u+\frac{\pi^{4}E\bar{A}}{4ml^{4}}u^{3}+\frac{c_{1}}{m}\dot{u}+\frac{3c_{3}\alpha }{2m}u^{2}\dot{u}=\frac{\pi^{2}\xi \bar{A}}{ml^{2}}uB\left(t\right)$$

where $\alpha =1+{\left(e_{0}a\right)}^{2}\pi^{2}/l^{2}$.

Let q=u and $p=\dot{u}$, Equation (2) can be expressed as follows

(3) $$\left\{ \begin{array}{l} \dot{q}=p\\ \dot{p}=-c_{1}^{\prime }q-c_{2}^{\prime }q^{3}-\left(2\eta +c_{3}^{\prime }q^{2}\right)p+eqB\left(t\right) \end{array}\right.$$

where $c_{1}^{\prime }=\frac{k_{w}l^{4}\alpha +\pi^{2}\xi \bar{A}Hl^{2}\alpha +\pi^{4}EI}{ml^{4}\alpha }$, $2\eta =\frac{c_{1}}{m}$, $c_{3}^{\prime }=\frac{3c_{3}\alpha }{2m}$ and $e=\frac{\pi^{2}\xi \bar{A}}{ml^{2}}$.

## 3. Nonlinear Dynamic Characteristics of Single-Walled Carbon Nanotubes

To a weakly nonlinear stochastic differential equation, there are many methods to obtain its approximate solution. However, the cubic nonlinear stiffness of this system here induces strongly nonlinear stiffness comparing with linear stiffness. The strong nonlinearity is also caused by large deformation. In this paper, a new method is developed to solve the dynamic response of this strongly nonlinear system.

Similar to any stochastic average method, the common nonlinear stochastic dynamic methods are based on the relationship between the system dynamic response and the system Hamiltonian function. To a weakly nonlinear stochastic system $\ddot{q}+c_{1}^{\prime }q+c_{2}^{\prime }q^{3}=0$, its Hamiltonian function can be shown as $\bar{H}=\frac{1}{2}p^{2}+\frac{1}{2}{c^{\prime }}_{1}q^{2}$, where $c_{1}^{\prime }$ is the linear stiffness. It implies that the system Hamiltonian function is only determined by the linear stiffness because the nonlinear stiffness is insignificant. However, to a strongly nonlinear stochastic system as that in Equation (3), its Hamiltonian function can be shown as

(4) $$\bar{H}=\frac{1}{2}p^{2}+\frac{1}{2}c_{1}^{\prime }q^{2}+\frac{1}{4}c_{2}^{\prime }q^{4}$$

From Equation (4), it is observed that the nonlinear stiffness coefficient $c_{2}^{\prime }$ also affects the system Hamiltonian function. It is practically difficulty to directly apply the stochastic average method because elliptic functions will appear in the Hamilton function solution. The energy function method is introduced to avoid this problem. An equivalent linear Hamiltonian function is established to take the place of Equation (4). The effect of the nonlinear stiffness to the original system Hamiltonian function can be considered as a modification to the new system natural frequency. Then, the new system Hamiltonian function can be shown as

(5) $$\bar{H}=\frac{1}{2}p^{2}+\frac{1}{2}kq^{2}$$

where $k=k\left(c_{1}^{\prime },c_{2}^{\prime }\right)=\omega^{2}$, ω is the new system natural frequency. Obviously, $\omega \neq \sqrt{c_{1}^{\prime }}$ and ω is affected by both the linear stiffness and nonlinear stiffness of the original system. If the expression of ω is determined, then the stochastic average method can be applied to solve the system dynamic response.

Let $\upsilon \left(q\right)=\frac{1}{2}c_{1}^{\prime }q^{2}+\frac{1}{4}{c^{\prime }}_{2}q^{4}$, we obtain that $\bar{H}=\frac{1}{2}p^{2}+\upsilon \left(q\right)$. q(t)=Acosφ+b, $p(t)=\dot{q}(t)$. According to the energy function method [45]

(6) $$q=q\left(\bar{H},\phi \right)=A\left(\bar{H}\right)\cos\phi +b\left(\bar{H}\right)$$

(7) $$\dot{q}=p=p(\bar{H},\phi )=\pm \sqrt{2\left[v\left(A\left(\bar{H}\right)+b\left(\bar{H}\right)\right)-v\left(A\left(\bar{H}\right)\cos\phi +b\left(\bar{H}\right)\right)\right]}$$

The boundary conditions of the energy function can be shown as

(8) $$\mathrm{For}\;\varphi =0,\;q=A\left(\bar{H}\right)+b\left(\bar{H}\right)\;\mathrm{and}\;p=0$$

(9) $$\mathrm{For}\;\varphi =\pi ,\;q=-A\left(\bar{H}\right)+b\left(\bar{H}\right)\;\mathrm{and}\;p=0$$

Thus,

(10) $$\upsilon \left(A\left(\bar{H}\right)+b\left(\bar{H}\right)\right)=\upsilon \left(-A\left(\bar{H}\right)+b\left(\bar{H}\right)\right)=\bar{H}$$

Since $\upsilon \left(q\right)=\frac{1}{2}c_{1}^{\prime }q^{2}+\frac{1}{4}c_{2}^{\prime }q^{4}$, we can see that υ(q) must be even, hence $b\left(\bar{H}\right)=0$ and $\upsilon \left(\bar{A}\right)=\bar{H}$. Then

(11) $$\frac{c_{1}^{\prime }}{2}A^{2}+\frac{c_{2}^{\prime }}{4}A^{4}=\bar{H}$$

(12) $$q=q\left(\bar{H},\phi \right)=A\cos\phi$$

(13) $$p=p\left(\bar{H},\phi \right)=A\sin\phi \sqrt{c_{1}^{\prime }+\frac{3c_{2}^{\prime }}{4}A^{2}+\frac{c_{2}^{\prime }}{4}A^{2}\cos2\phi }$$

Thus, the system natural frequency is

(14) $$\omega =\sqrt{c_{1}^{\prime }+\frac{3c_{2}^{\prime }}{4}A^{2}}$$

where A is determined by Equation (11).

At this stage it is possible to apply the stochastic average method to solve the system dynamic response. According to the theory of the quasi-nonintegrable Hamiltonian system, the Hamiltonian function converges weakly in a probability sense to a one-dimensional Ito diffusion process. The averaged Ito equation about the Hamiltonian function can be shown as

(15) $$d\bar{H}=\bar{m}\left(\bar{H}\right)+\bar{\sigma }\left(H\right)dB\left(t\right)$$

where B(t) is the standard Wiener process and $\bar{m}\left(\bar{H}\right)$ and $\bar{\sigma }\left(\bar{H}\right)$ are the drift and diffusion coefficients of Ito stochastic process, which can be obtained through the stochastic averaging method. Then

(16) $$\bar{m}\left(\bar{H}\right)=\frac{De^{2}}{\omega^{2}}\bar{H}-\frac{2\sqrt{2}c_{1}^{\prime }}{3\pi \omega^{2}}{\bar{H}}^{\frac{3}{2}}+\frac{4\sqrt{2}c_{2}^{\prime }}{5\pi \omega^{2}}{\bar{H}}^{\frac{5}{2}}-\frac{c_{3}}{2\omega^{4}}{\bar{H}}^{3}$$

(17) $${\bar{\sigma }}^{2}\left(\bar{H}\right)=\frac{De^{2}}{\omega^{2}}{\bar{H}}^{2}$$

The averaged Fokker-Planck-Kolmogorov equation (FPK equation) [46] of Equation (15) is

(18) $$\frac{\partial f}{\partial t}=-\frac{\partial }{\partial \bar{H}}\left[\bar{m}\left(\bar{H}\right)f\right]+\frac{1}{2}\frac{\partial^{2}\left[{\bar{\sigma }}^{2}\left(\bar{H}\right)f\right]}{\partial {\bar{H}}^{2}}$$

where f is the stationary probability density (SPD) function of the system response, and

(19) $$f\left(\bar{H}\right)=\tilde{A}{\bar{H}}^{\frac{2\eta }{\left(2+3\sqrt{2}\right)De^{2}}}\exp\left(-\frac{4\sqrt{2}c_{1}^{\prime }}{3\pi De^{2}\omega^{2}}{\bar{H}}^{\frac{1}{2}}+\frac{8\sqrt{2}c_{2}^{\prime }}{5\pi De^{2}\omega^{2}}{\bar{H}}^{\frac{3}{2}}-\frac{c_{3}^{\prime }}{2De^{2}\omega^{4}}{\bar{H}}^{2}\right)$$

where $\tilde{A}$ is a normalization constant.

**Figure 2:**
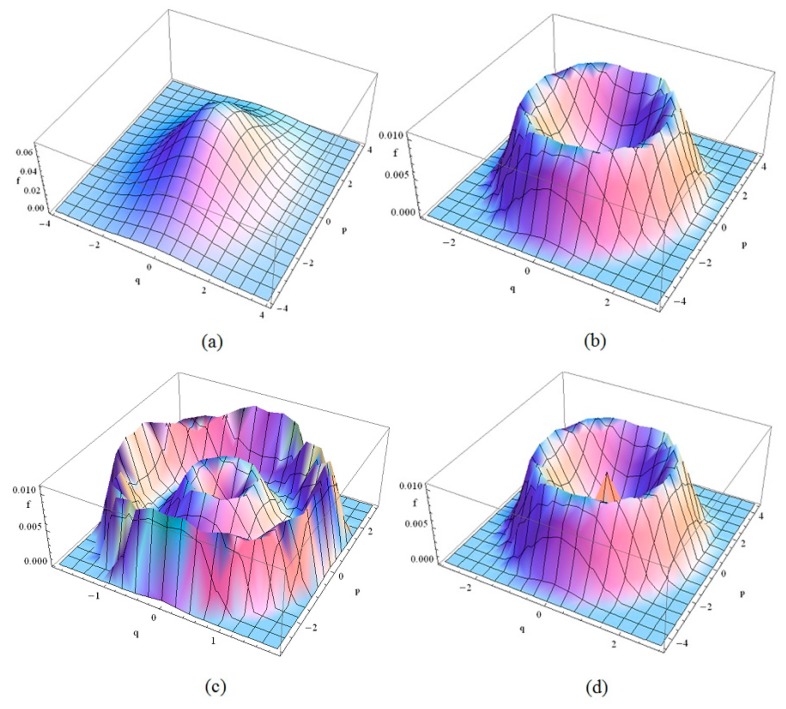
Stationary probability density of the system response. (**a**) D=0.1; (**b**) D=0.4; (**c**) D=0.6; (**d**) D=0.8.

SPD numerical simulation of the system response is shown in Figure 2, where $c_{1}^{\prime }=1$, $c_{2}^{\prime }=0.3$, η=0.5, $c_{3}=0.1$ and e=0.6.

From Figure 2, it is observed that

(1) For small D, the steady-state probability density of H=0 is the maximum which implies that the system may be stable at the original point and the system motion is a slight vibration near the balance point (0, 0) in a probability sense. With increasing D, a crest occurs in the SPD map and the system motion is periodic in a probability sense which may cause system vibration and reduce the system characteristics.
(2) With further increasing of D, two loops occur in the SPD map. It implies that the system motion has two possible occurrences and each of them is periodic. The system response can jump from one periodic motion to another under an external excitation, which in turn causes the mutation of vibration amplitude.
(3) For D at a high level, a crest and a loop occur in the SPD map. It implies that the system motion has two possible occurrences, one is a small vibration near the balance point (0, 0) and the other is a periodic motion. The system response can jump from the small vibration to the periodic motion under an external excitation. The vibration amplitude of the periodic motion is large than that of the small vibration.
(4) In summary, the stochastic magnetic field intensity D affects significantly the system response. An increase in D may lead to an increasingly unstable system and instability may by reduced with further increasing of D. It implies there exists a value D has the maximum influence on the system stability and it is called the stochastic resonance.

## 4. Safe Basin and Reliability

There are heteroclinic orbits in the system, which can be expressed as follows:

(20) $$\bar{q}\left(t\right)=\pm \sqrt{-\frac{c_{1}}{c_{2}}}\tanh\left(\frac{\sqrt{2c_{1}}}{2}t\right)$$

(21) $$\bar{p}\left(t\right)=\pm \frac{c_{1}}{2}\sqrt{-\frac{2}{c_{2}}}\sec h^{2}\left(\frac{\sqrt{2c_{1}}}{2}t\right)$$

**Figure 3:**
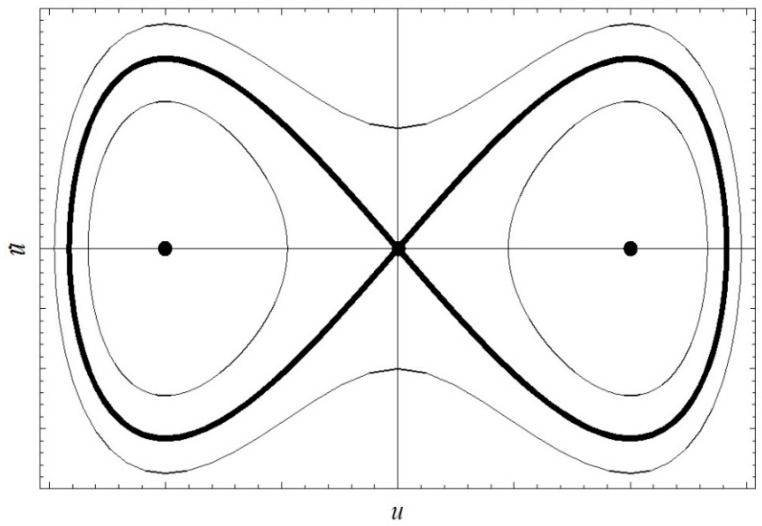
System heteroclinic orbits.

The heteroclinic orbits of the system are illustrated in Figure 3.

**Figure 4:**
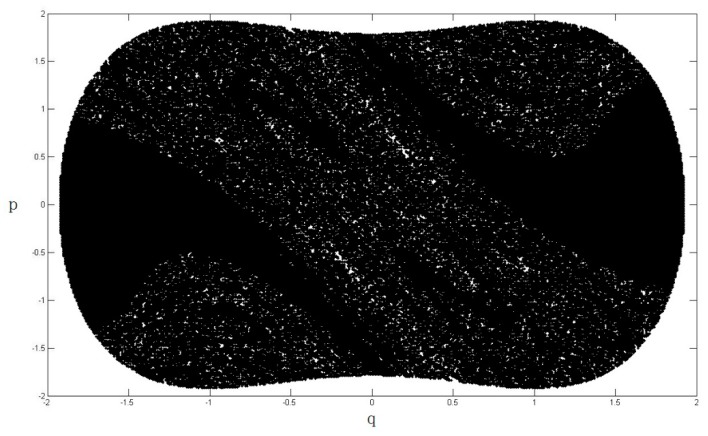
Safe basin of the system when e = 0.2.

**Figure 5:**
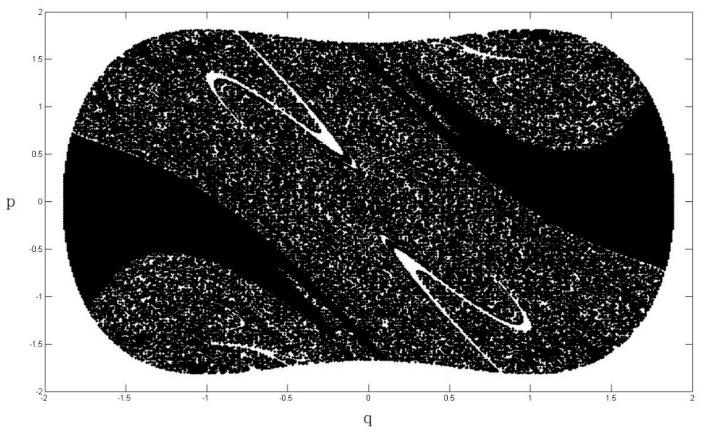
Safe basin of the system when e = 0.4.

**Figure 6:**
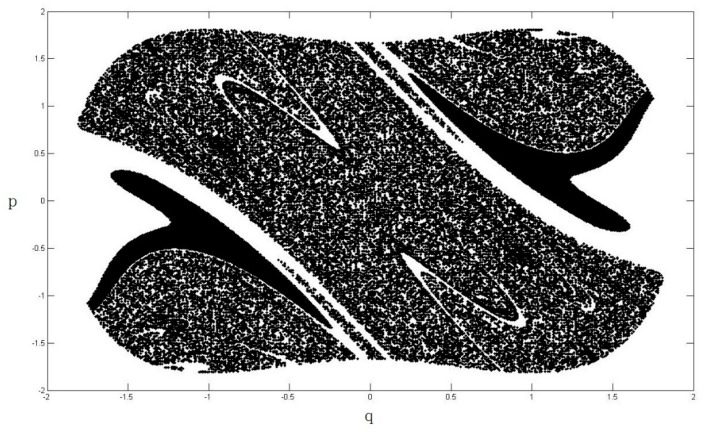
Safe basin of the system when e = 0.6.

**Figure 7:**
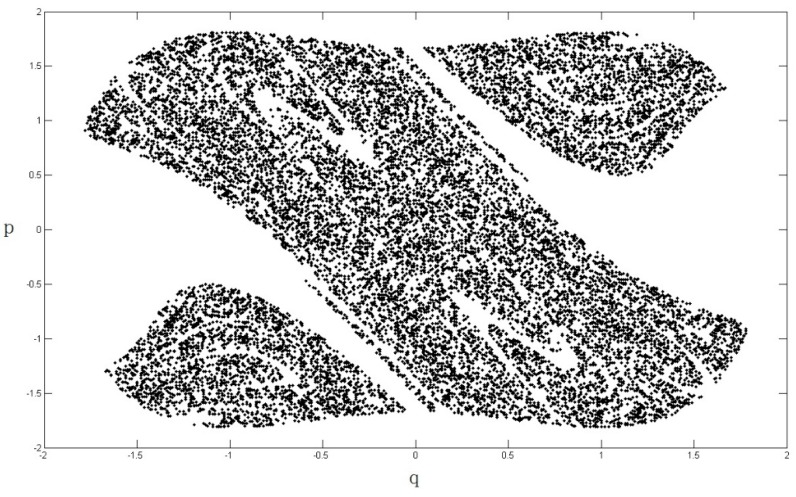
Safe basin of the system when e = 0.8.

For the stochastic system in Equation (3), the boundary of its safe basin can be determined by the stochastic Melnikov integration as follows:

(22) $$M\left(t_{1}\right)=\int_{-\infty }^{+\infty }\bar{p}\left[-2\eta \bar{p}+e\bar{q}\varsigma \left(t_{1}-t\right)\right]dt=-I+z\left(t_{1}\right)$$

where $\bar{p}$ and $\bar{q}$ are the heteroclinic orbits, which are shown in Equations (20) and (21). The term −I in Equation (22) represents the mean of the Melnikov process due to a damping force, $-I=\int_{-\infty }^{+\infty }-2\eta {\bar{p}}^{2}dt$; and $z\left(t_{1}\right)$ denotes the random portion of the Melnikov process due to the stochastic noise ς(t), $z\left(t_{1}\right)=\int_{-\infty }^{+\infty }e\bar{p}\bar{q}\varsigma \left(t_{1}-t\right)dt$. The stochastic Melnikov integration $M\left(t_{1}\right)=0$ means that the motion system become chaotic, hence we can determine the chaotic boundary of the system according to $M\left(t_{1}\right)=0$. The variation in the safe basin of the system Equation (3), which is subjected to a stochastic excitation, is illustrated in Figure 4, Figure 5, Figure 6 and Figure 7.

From Figure 4, Figure 5, Figure 6 and Figure 7, it is clear that the area of safe basin decreases significantly with increasing parameter e. $e=\frac{\pi^{2}\xi \bar{A}}{ml^{2}}$, the parameter e is directly related to the magnetic field permeability ξ, all the parameters are introduced in Section 2. Therefore it can be concluded that the magnetic field permeability plays an important role in the system safe basin.

The safe basin area describes the system reliability qualitatively. To discuss the system reliability quantitatively, the concept of first-passage is introduced to describe the system reliability. The background Kolmogorov equations (BK equations) of the reliability function and the probability density of the first-passage time can be shown as

(23) $$\frac{\partial R}{\partial t}=\bar{m}\left(\bar{H}\right)\frac{\partial R}{\partial \bar{H}}+\frac{1}{2}{\bar{\sigma }}^{2}\left(\bar{H}\right)\frac{\partial^{2}R}{\partial {\bar{H}}^{2}}$$

(24) $$P\left(T|{\bar{H}}_{0}\right)=-\frac{\partial R\left(t|{\bar{H}}_{0}\right)}{\partial t}|t=T$$

where R is the reliability function of the system, T is first-passage time, P is the probability density of the first-passage time. The initial condition is

(25) $$R\left({\bar{H}}_{0},0\right)=1,\;{\bar{H}}_{0}\in \Gamma ,\;\mathrm{when}\;t=0$$

The boundary conditions are

(26) $$R\left({\bar{H}}_{0},t\right)=0,\;\mathrm{when}\;{\bar{H}}_{0}=\Gamma$$

(27) $$\frac{\partial R}{\partial t}=\bar{m}\left(\bar{H}\right)\frac{\partial R}{\partial \bar{H}},\;\mathrm{when}\;{\bar{H}}_{0}=0$$

**Figure 8:**
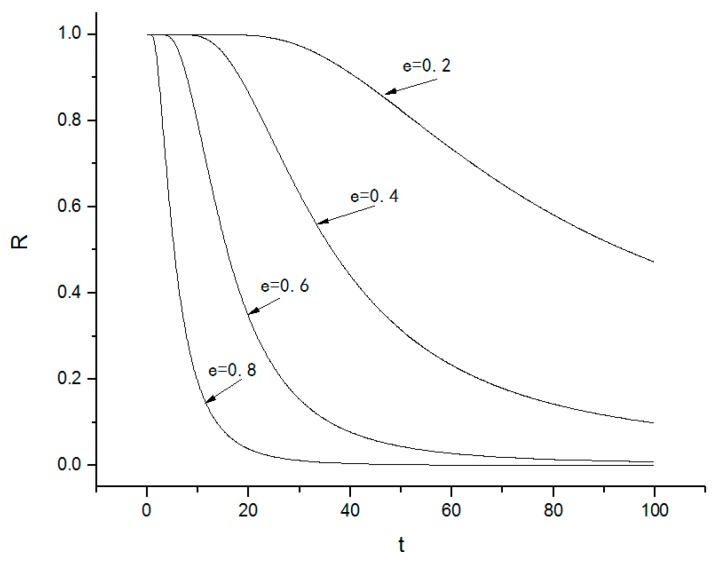
System reliability.

**Figure 9:**
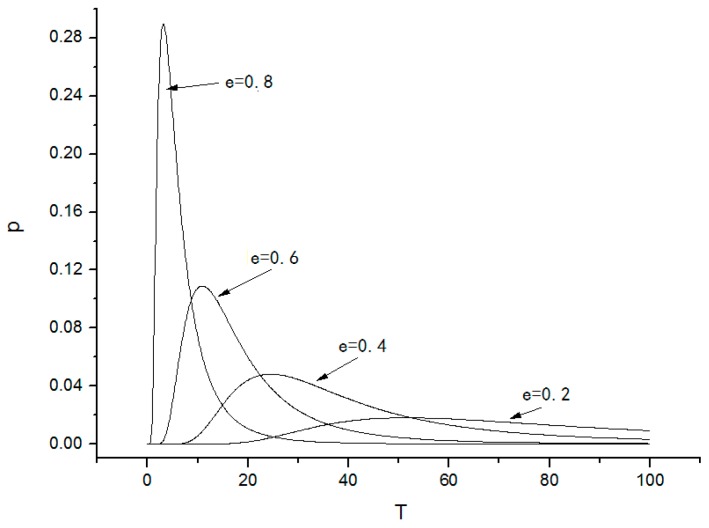
Probability density of the first-passage time.

Numerical simulations of the system reliability function and the probability density of the first-passage time are shown in Figure 8 and Figure 9.

From Figure 8 and Figure 9, it is observed that

(1) The system reliability function $R\left(\bar{H},t\right)$ decreases with increasing time, which indicates that the probability of the system to stay in the safe basin becomes increasingly smaller and the probability of damage to the system increases. If the parameter e is large enough, the system reliability will decrease quickly; if the parameter e is small, the system reliability decreases slowly. Thus, the parameter e significantly affects the system reliability.
(2) The probability density of first-passage time increases with time. First-passage means that the leaving of the system from the safe area and it causes system instability. There exists a peak in the probability density of the first-passage time that corresponds to the time when the system leaves the safe basin.

## 5. Conclusions

Theoretical analysis and numerical simulations show that stochastic resonance occurs when varying the random magnetic field intensity. It is concluded that the boundary of safe basin has fractal characteristics and the area of safe basin decreases when the intensity of the magnetic field permeability increases. A conclusion can be deduced that the magnetic field plays a significant role in the system vibration response: the deterministic part of magnetic field affects the system safe basin while the stochastic part induces stochastic resonance.

Both stochastic resonance and safe basin decreasing can reduce the stability of Nanotubes and do harm to the life-time dilatation of NEMS. The stability and safety can be obtained by control strategy keeping away from stochastic resonance and safe basin decreasing. The results of this paper are helpful for the application of Nanotube in engineering.
